# Building a National Interoperable Rare Eye Disease Data Warehouse: Methodological Framework and Implementation Report From the French Rare Eye Disease Database (FREDD) Initiative

**DOI:** 10.2196/99206

**Published:** 2026-08-25

**Authors:** Camille Beluffi Marin, Marilyne Oswald, Laura Ratenet, Matthieu Stoll, Kirsley Chennen, Nathalie Chabin, Julie Hubert, Grégoire Rey, Guylène Le Meur, Vasily Smirnov, Isabelle Meunier, Isabelle Audo, Hélène Dollfus

**Affiliations:** 1 Laboratoire de Génétique Médicale, 1 rue Eugène Boeckel, Strasbourg, Grand Est, 67000, France, 33 368853065; 2 FSMR SENSGENE, Centre de Référence Pour les Affections Rares en Génétique Ophtalmologique (CRMR CARGO), Hôpitaux Universitaires de Strasbourg, Strasbourg, Grand Est, France; 3 Laboratoire de Génétique Médicale, 1 rue Eugène Boeckel, Strasbourg, Grand Est, 67000, France; 4 France Cohortes, Inserm, Ined, Sorbonne Université, Université Paris Cité, Université Paris Saclay, Paris, France; 5 Nantes University, ophthalmology department Nantes University Hospital, Inserm, TARGET, Nantes, Pays de la Loire, France; 6 Exploration de la Vision et Neuro-Ophtalmologie, CHU de Lille, Lille, Hauts-de-France, France; 7 National Reference Centre for Inherited Sensory Diseases, University of Montpellier, Montpellier, Occitanie, France; 8 Centre Hospitalier National d'Ophtalmologie des Quinze-Vingts, Centre de Référence Maladies Rares REFERET and INSERM-DGOS CIC 1423, Paris, Île-de-France, France; 9 FSMR SENSGENE, Centre de Référence Pour les Affections Rares en Génétique Ophtalmologique (CRMR CARGO), Institut de Génétique Médicale d'Alsace (IGMA), ERN-EYE, Hôpitaux Universitaires de Strasbourg, Strasbourg, France

**Keywords:** rare eye diseases, retinitis pigmentosa, health data warehouse, secondary use of health data, clinical research, data interoperability, data governance, regulatory bodies, inherited retinal dystrophies

## Abstract

**Background:**

Rare eye diseases are characterized by low prevalence, clinical heterogeneity, and fragmented data collection, which limit the reliability of analysis results and multicenter research. In France, the development of health data warehouses is strictly regulated by national data protection authorities. While international initiatives aim to harmonize rare disease registries, capturing hyperspecialized, multimodal clinical records and ophthalmology-specific imaging within a fully compliant and sustainable infrastructure remains a major operational challenge.

**Objective:**

This study aims to describe the methodological framework, regulatory implementation, and early operational outcomes of FREDD (French Rare Eye Disease Database), a national interoperable health data warehouse dedicated to rare eye diseases, and to analyze its key success factors, technical bottlenecks, and long-term financial sustainability models.

**Methods:**

Developed under the oversight of the French National Institute of Health and Medical Research (Institut national de la santé et de la recherche médicale; INSERM), the warehouse implements a centralized 3-layer architecture encompassing data collection, processing, and research reuse environments. The dataset extends the national minimum rare disease dataset with detailed ophthalmology variables structured through a dynamic electronic case report form, as well as a dedicated collection of ophthalmic images. Interoperability is achieved using international domain ontologies and a custom parsing tool (FREDDEX) designed to automatically prefill clinical data from the existing French national rare disease registry. Centralized monitoring, data curation, and cross-center quality tracking are driven by a dedicated in-house dashboard (FREDDIn [FREDD Insights]), while unstructured retinal images are processed through a standardized pseudonymization and human-verified validation pipeline.

**Implementation (Results):**

Official regulatory authorization was obtained in April 2024, and active data collection began in March 2025 across 5 pilot expert centers, focusing its initial phase on inherited retinal dystrophies, primarily retinitis pigmentosa. Over a 1-year period, the warehouse successfully integrated clinical data from 1649 patients—representing 20.3% of all retinitis pigmentosa cases registered nationally—and accumulated more than 10,000 ophthalmic images. The centralized dashboard maintained an overall data inconsistency rate of approximately 7%, chiefly reflecting logical dependencies. Among the included patients, 36.7% (605/1649) patients had associated imaging data, with fundus photography being the most widely available modality (768/1649, 46.6%).

**Conclusions:**

The implementation of this database demonstrates that a highly specialized, multimodal health data warehouse can be successfully deployed in a highly regulated environment when regulatory anticipation, governance formalization, and technical flexibility are addressed in parallel. While initial development was supported by institutional grants, long-term sustainability will require a structured cost-recovery model. Future milestones will focus on expanding center coverage nationwide, refining image annotation frameworks for AI readiness, and deploying automated export pathways toward European registries.

## Introduction

### Context

#### The French Rare Disease Ecosystem

Over the past 2 decades, successive French national plans for rare diseases (PNMRs) have structured national rare disease care and research. During PNMR2 (2011‐2016) [1], the Filières de Santé Maladies Rares (FSMR) network was established to coordinate expert centers, while the National Registry for Rare Diseases (Banque Nationale de Données Maladies Rares [BNDMR]) centralized standardized clinical data through BaMaRa (Base maladies rares), a national data collection system supporting both clinical management and secondary data use [2-4]. PNMR3 (2018‐2022) [5] further strengthened structured data collection by integrating BNDMR into the Health Data Hub (HDH) [6] to facilitate secure linkage with National Health Insurance data. Within this framework, the SENSGENE FSMR [7] coordinates 91 expert centers dedicated to sensory diseases. PNMR4 (2025‐2030, “From Territories to Europe”) [8] now aims to further develop health data resources, biobanking, and international interoperability.

This ecosystem operates under the oversight of the French data protection authority (CNIL, Commission nationale de l’informatique et des libertés [9]) and complies with the French Data Protection Act (Loi Informatique et Libertés [10]) and the European General Data Protection Regulation (GDPR [11]).

#### European Integration: ERN-EYE and REDgistry

At the European level, rare disease expertise is coordinated through European Reference Networks (ERNs). For rare eye diseases (REDs), ERN-EYE [12] connects over 60 specialized centers across 24 member states to facilitate cross-border expertise and collaborative research. The REDgistry [13], launched in 2022, provides a European registry framework to harmonize RED data collection and enable multicentric analyses. These initiatives align with the emerging European Health Data Space (EHDS [14]), which aims to support secure and interoperable reuse of health data across the European Union.

#### The Digital Health and AI Opportunity in Rare Diseases

National and European platforms, such as the BNDMR and REDgistry, have become pivotal for large-scale, data-driven multicenter research, cohort building, and AI applications [15-22]. However, while broader initiatives such as RD-Connect [23], BBMRI-ERIC [24], EPIRARE [25], and the ERNs have established foundational frameworks for data standardization and cross-border sharing, they typically operate at a generic rare disease level or focus primarily on biological sample governance and federated access.

### Identified Gap

Despite this flourishing national and European context, a critical limitation remains. While the BNDMR collects data across all rare diseases, it is primarily designed for care coordination and epidemiological monitoring, thus lacking ophthalmology-specific details such as imaging data or visual function measurements. Conversely, REDgistry aggregates data at the European level but cannot capture the full spectrum of national patient cohorts, potentially overlooking local research priorities and multicenter project feasibility. To address these limitations, comprehensive national rare disease registries should be integrated with disease-specific European infrastructures, creating a continuum from routine clinical data collection to highly specialized ophthalmic research while maintaining international interoperability.

### Aims and Objectives

To address this gap, clinicians from the SENSGENE network created FREDD (French Rare Eye Disease Database) [26], a dedicated health data warehouse (HDW) designed to centralize, structure, and enrich clinical data from RED patients across multiple expert centers. FREDD is intentionally designed to follow, as closely as possible, existing and widely adopted principles of rare disease data infrastructures in terms of technical architecture, interoperability standards, and data governance models to ensure alignment with established national and European ecosystems. FREDD aims to provide a nationally coordinated repository for RED data; enable national multicenter research, cohort building, and longitudinal studies; facilitate interoperability with the French BNDMR, ERN-EYE, and the emerging EHDS; and support the development of new diagnostic and therapeutic strategies tailored to RED.

**Figure 1.**
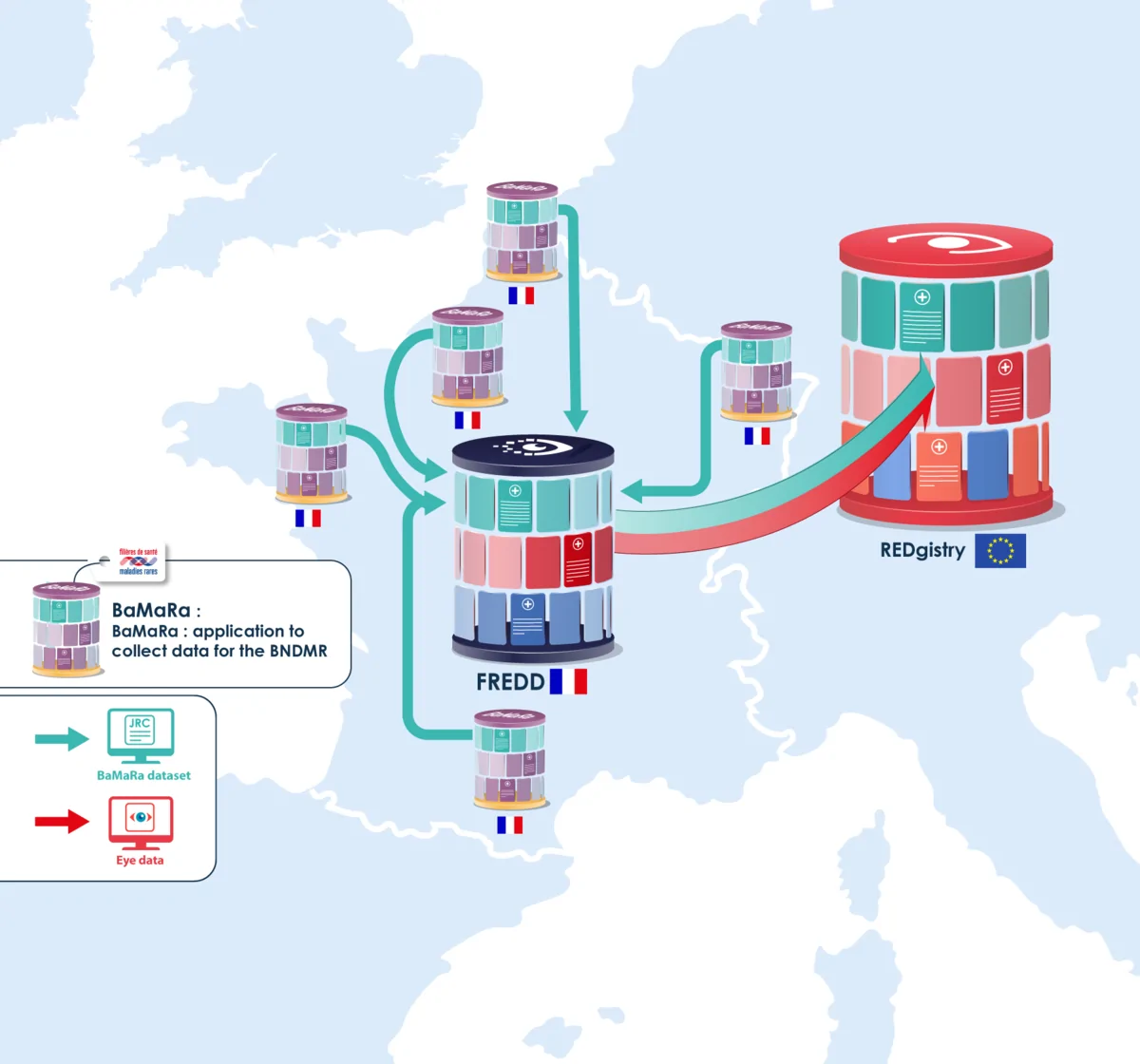
The rare eye disease ecosystem at the national and international levels. BaMaRa (Base maladies rares) and the BNDMR (Banque nationale des maladies rares) are French databases funded by the French Ministry of Health, and REDgistry is publicly funded through the European Commission. FREDD (French Rare Eye Disease Database) represents the bridge between the national and European ecosystems.

This multiscale articulation—from local clinical data collection to national structuring and European integration—is illustrated in Figure 1.

This manuscript presents the concrete deployment of this national infrastructure, detailing its methodological framework while providing a critical appraisal of both the enabling factors and the operational challenges encountered during its initial phases. To ensure a rigorous and standardized presentation of our system architecture, governance, and deployment outcomes, the manuscript has been formatted as an implementation report and adheres to the iCHECK-DH reporting framework [27].

## Methods

### Ethical Considerations

This article describes the development and implementation of the FREDD database and the assessment of its data collection and integration processes. This did not involve a research project using FREDD data for secondary analysis or the generation of new clinical research findings. The FREDD database has been authorized by the French data protection authority (CNIL) for health data processing under decision DT-2024-008 dated April 6, 2024. Access to FREDD data for secondary research use is subject to a separate governance and data access procedure.

### Blueprint Summary

The CNIL HDW reference framework [28] underpins FREDD’s design and operation, guiding both its technical architecture and regulatory implementation. It defines more than 100 organizational, technical, and legal requirements across 12 domains. A data protection impact assessment (DPIA) serves as the core regulatory document demonstrating compliance.

### Technical Design

To support multicenter data collection, postcollection quality control, and secure research reuse, FREDD relies on a centralized 3-layer HDW architecture:

First, the collection layer uses a dedicated electronic case report form (eCRF) developed with SKEZIA, a compliant and cost-effective commercial platform developed by SKEZI [29], for standardized clinical data capture and early pseudonymization at participating sites level. Second, the storage and processing layer is hosted within the French National Institute of Health and Medical Research (Institut national de la santé et de la recherche médicale; INSERM) France Cohortes infrastructure [30], where clinical datasets, genetic data, and high-volume imaging batches are securely ingested, harmonized, and curated. Third, the research layer provides project-specific, isolated virtual environments for governance-approved secondary data analysis under strict access permissions.

**Figure 2.**
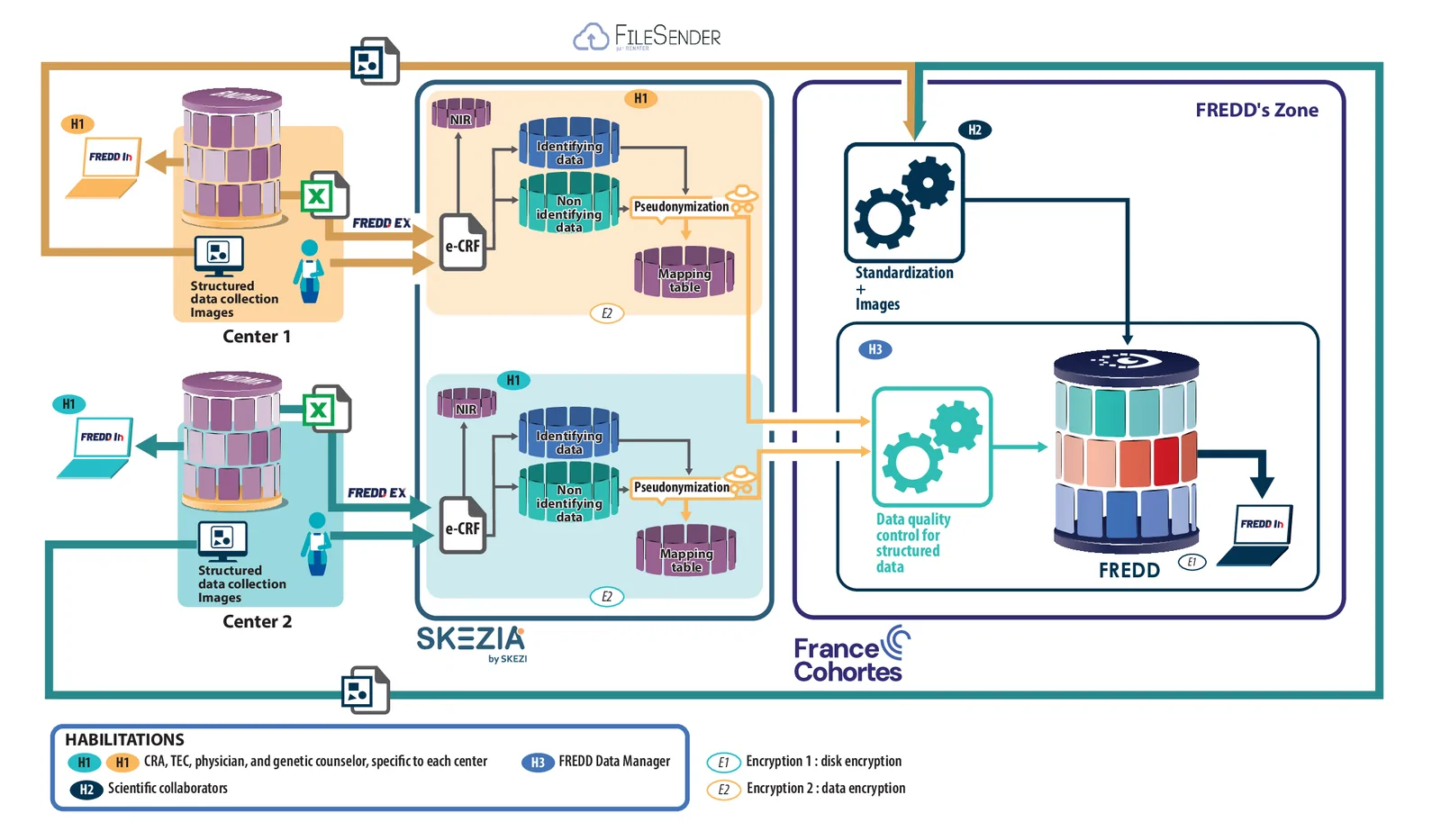
Diagram of the FREDD (French Rare Eye Disease Database) data life cycle within the information system, illustrating the different data processing steps and the measures taken to ensure data security and confidentiality, in accordance with the national regulatory framework and guidelines for health data warehouses. The diagram highlights the data life cycle from the collection layer, managed within the SKEZIA environment, to the data quality, storage, and historization layer in the France Cohortes environment. It also specifies who can access each type of data depending on their role. CRA: clinical research associate; eCRF: electronic case report form; FREDDIn: FREDD-Insight; TEC: Clinical Research Technician.

Data transfers between collection and processing environments currently rely on standardized, semiautomated batch exports via FileSender [31]. Access control follows the principle of least privilege, enforced through a formalized data access matrix. The overall system architecture and end-to-end data life cycle are illustrated in Figure 2. Granular technical specifications are detailed in Multimedia Appendix 1.

### Target

While FREDD is designed to ultimately aggregate data for all patients affected by any RED nationwide, the initial pilot phase implements a progressive and controlled deployment strategy. To validate the infrastructure, evaluate the warehouse’s scientific value, and assess center engagement and adoption of the new data-sharing workflows, initial data collection focuses on a selected group of pilot centers and restricts its clinical scope to inherited retinal dystrophies (IRDs). Within this broader IRD population, particular emphasis was placed on retinitis pigmentosa (RP), which represented the largest patient cohort available for early evaluation.

### Data

#### Structured Data

In line with regulatory requirements, FREDD captures only variables strictly necessary for its research objectives. The dataset encompasses up to 447 variables across 13 categories, including administrative data, clinical and genetic diagnoses, disease history, visual function, treatments, disability, clinical protocols, and imaging deposits. To ensure baseline research completeness, a maximum of 153 (34.2%) variables are designated as mandatory. Crucially, this framework is dynamic rather than static: field display and mandatory status adapt conditionally to each patient’s clinical pathway, preventing clinical team overburden. Detailed variable distributions and complex categories are provided in Multimedia Appendices 2 and 3.

#### Imaging Data

A distinctive feature of FREDD is the collection of retinal imaging data, including fundus photography, autofluorescence, and optical coherence tomography (OCT) single cross-sectional scans (B-scans) rather than full 3D volumes, which are essential for accurate diagnosis. To overcome heterogeneity across acquisition devices and formats while preserving structural features for AI research, images undergo a standardized 4-step processing pipeline: (1) resizing and color harmonization, (2) pseudonymized DICOM (Digital Imaging and Communications in Medicine) object reconstruction, (3) hybrid automated text detection and human-verified screening for embedded identity leaks, and (4) secure deletion of raw source files after validation.

To mitigate any risk of retaining directly identifying information, original source images are securely deleted from the processing environment immediately after processing. Consequently, only images that successfully complete this full pipeline are permanently stored and uploaded into the final FREDD warehouse.

A step-by-step description of the image-processing pipeline is detailed in Multimedia Appendix 4.

#### Structured Data Management and Monitoring

To ensure data accuracy and intercenter harmonization from the point of entry, standardized data entry procedures are disseminated to all participating centers. These procedures provide detailed operational definitions for each variable, standardized coding rules, and guidance for handling ambiguous clinical situations, thereby minimizing interoperator variability and reducing downstream data cleaning requirements.

Postcollection curation, quality control, and tracking are driven by FREDDIn (FREDD-Insight), a centralized, in-house dashboard developed via Python scripts. FREDDIn automatically performs data standardization, temporal consistency checks, anomaly detection, and patient deduplication. It is deployed both locally, to support participating centers, and centrally within France Cohortes for national quality monitoring.

### Interoperability

**Figure 3.**
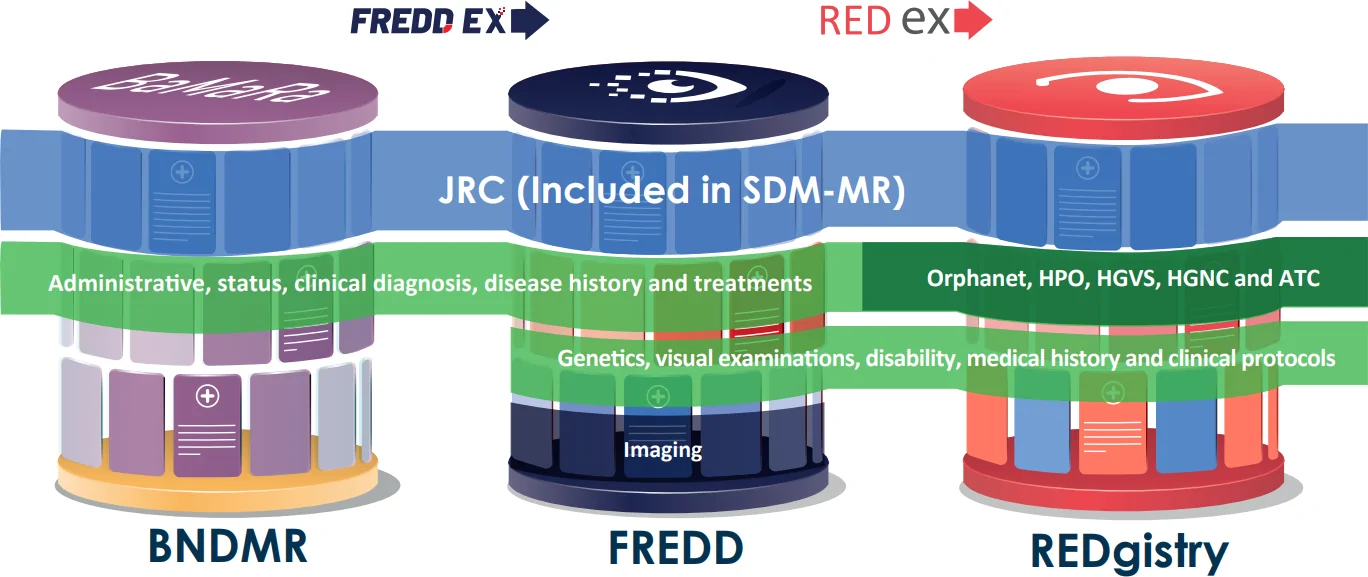
Diagram illustrating FREDD’s (French Rare Eye Disease Database) architectural role as a technical and semantic bridge between the French national rare disease registry (Banque Nationale de Données Maladies Rares [BNDMR] and Base Maladies Rares [BaMaRa]) and the European registry (REDgistry). The blue bar highlights the European Joint Research Centre (JRC) core variables shared across all repositories. Green bars delineate the specific clinical data categories captured and the international used for standardized semantic mapping. The dark blue bar highlights hyperspecialized ophthalmology extensions and imaging data unique to FREDD. Red arrows highlight automated data mapping pipelines powered by custom parsing tools: FREDDEX for data ingestion from BaMaRa, and REDDEX for data exports toward REDgistry. ATC: Anatomical Therapeutic Chemical Classification; HGNC: HUGO Gene Nomenclature Committee; HGVS: Human Genome Variation Society; HPO: Human Phenotype Ontology; JRC: joint research center; SDM-MR: Set de Données Minimum–Maladies Rares.

To maximize semantic and technical interoperability, the dataset is built upon national and international standards. As illustrated in Figure 3, the FREDD data model integrates the French BNDMR core framework (via the Set de Données Minimum–Maladies Rares [32]) and overlaps with the European REDgistry schema, which is conceptually derived from the Joint Research Centre common data elements [33,34]. To address the limitations of these generic rare disease frameworks, FREDD extends this core model with ophthalmology-specific variables and imaging data.

To significantly reduce manual data entry, FREDD uses FREDDEX [35], a dedicated parsing and mapping tool that automatically retrieves clinical data already available in each patient’s BaMaRa record upon prospective enrollment into FREDD. Among the 447 variables defined in the FREDD dataset model, 281 are potentially retrievable from BaMaRa, corresponding to a theoretical maximum prefill capacity of 62.9%. This represents the upper interoperability potential of the pipeline, while the effective completion rate remains dependent on source data completeness and quality. Considering duplicated or repeated variable structures within FREDD (eg, main and additional diagnoses), these 281 target variables correspond to 83 unique BaMaRa source variables that can be extracted and mapped to FREDD. Among these source variables, 23 are mandatory BaMaRa fields and are therefore systematically available, providing a guaranteed baseline availability of 28% of BaMaRa-derived variables.

FREDDEX extracts BaMaRa exports, maps the variables to their corresponding eCRF fields, and enforces international domain-specific ontologies and standards, including disease classification using Orphanet (ORPHA) codes [36], phenotypic annotation using Human Phenotype Ontology (HPO) terms [37], and genetic nomenclature following HUGO Gene Nomenclature Committee (HGNC) [38] and Human Genome Variation Society [39] standards for gene and variant identification.

Furthermore, a complementary tool currently under development, REDDEX, leverages these identical interoperability principles to enable automated, structured data transfer from FREDD back toward the European REDgistry infrastructure. REDDEX is currently undergoing production testing and is scheduled to be fully operational by late 2026.

### Participating Entities

FREDD is an initiative developed and led by the SENSGENE rare disease network, which drove the inception of the project. Administratively and legally, FREDD operates under the responsibility of the INSERM [40], acting as the central data controller.

To demonstrate the feasibility of the architecture and gather early validation data, FREDD is currently deployed across 5 pilot expert centers within the SENSGENE network, which actively feed data into the warehouse:

1. CARGO (Rare Disease Center for Ocular Genetics) at Strasbourg University Hospital
2. REFERET (Rare Disease Center for Hereditary Retinal Dystrophies) at National Ophthalmology Hospital (Quinze-Vingts), Paris
3. REFERET at Nantes University Hospital
4. MAOLYA (Rare Disease Center for Severe Myopia, Neuro-Ophthalmology, and Hereditary Optic Neuropathies) at Lille University Hospital
5. MAOLYA at Montpellier University Hospital

### Regulatory Aspects, Governance, and Phased Deployment

The FREDD legal framework governs all data life cycle operations through 3 central pillars:

First, governance and data reuse, where a steering committee oversees strategic policies twice yearly, data access for reuse requires a formal review of all research proposals by the scientific and ethics committee, and researchers receive variable-specific completeness reports before extraction to guide data quality strategies.

Second, legal and ethical formalization, where operational standards are defined in a governance charter approved in 2025, complemented by data-sharing agreements with participating centers.

Third, patient rights and transparency, where to ensure full GDPR and CNIL compliance, patients are informed through a dedicated transparency portal and physical brochures in clinical centers, with accessible formats specifically provided for visually impaired patients.

Following the DPIA submission in December 2022, FREDD obtained official CNIL authorization in April 2024. The collection layer became operational and compliant in March 2025, allowing structured data entry to begin while the full France Cohortes infrastructure finalized compliance in November 2025. During this interim phase, collected datasets remained confined to the collection layer; imaging integration was initiated thereafter. The detailed timeline is available in Multimedia Appendix 5.

### Budget Planning and Sustainability

The launch and development of FREDD were made possible by the RaReTiA project (ANR-21-PMRB-0009), initiated in October 2022. This project was funded through a specific call for proposals launched by the French Ministries of Health and Research under PNMR3 and the France 2030 program, titled Accelerating Research and Innovation on Rare Diseases through Databases.

This dedicated budget successfully covered the entire “build” phase of FREDD, as well as its initial deployment and the beginning of the “run” phase. However, because this institutional funding is time-limited, it does not cover the expenses associated with FREDD beyond the scope and duration of the RaReTiA project.

## Implementation (Results)

### Coverage

**Figure 4.**
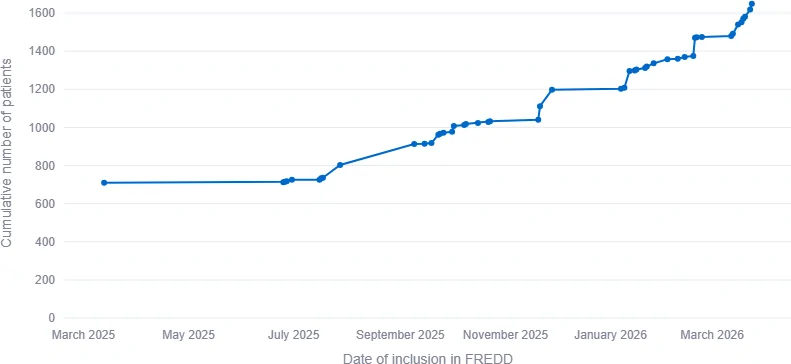
Evolution of patient inclusions in FREDD (French Rare Eye Disease Database) from the start of data collection in March 2025 to March 2026, representing 1 year of data acquisition.

Over a 1-year data acquisition period spanning from March 2025 to March 2026, FREDD successfully included a total of 1649 patients across the 5 participating pilot centers, demonstrating a steady and robust inclusion dynamic (Figure 4). Image collection was initiated in November 2025, and more than 10,000 ophthalmic images were subsequently integrated into the warehouse during the study period.

Among the 1649 included IRD patients, 763 (46.3%) had a diagnosis of RP (ORPHA:791). According to the February 2026 BNDMR national report [41], 3764 patients with RP have been registered nationwide since the deployment of the registry. Therefore, within its first year of implementation and across only 5 expert centers, FREDD has already enriched data corresponding to 20.3% (763/3764) of the national BNDMR RP cohort. These patients originated from existing BaMaRa records and were further characterized through the systematic collection of disease-specific ophthalmological variables, multimodal imaging data, centralized quality control procedures, and research-oriented governance. This rapid enrichment of a substantial proportion of the national RP cohort highlights the potential of disease-specific data infrastructures to transform routine rare disease records into structured, research-ready resources.

### Outcomes

**Figure 5.**
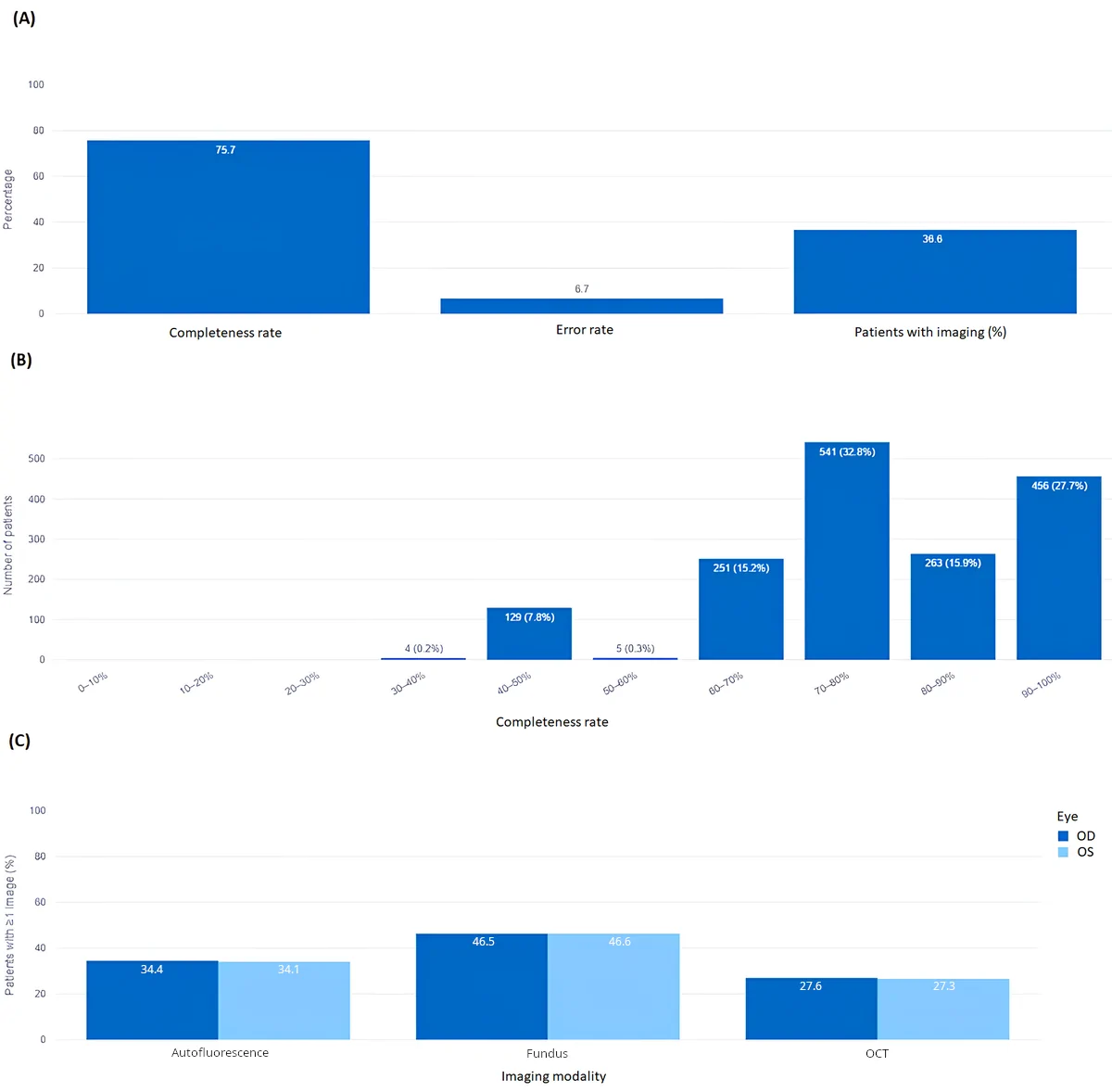
Overview of data quality, completeness scores, and ophthalmic imaging coverage. The charts provide a descriptive evaluation of the integrated dataset during the first year of data collection. (A) Overall data quality metrics showing the mean completeness rate, error rate, and the percentage of the cohort with associated imaging data. (B) Distribution of the patient cohort (N=1649) across categorical data completeness score ranges, detailing both absolute counts and percentages for each bin. (C) Proportional imaging coverage across the primary modalities, stratified by eye lateralization (right eye [OD] vs left eye [OS]). OCT: optical coherence tomography.

Overall, the FREDD database demonstrates high completeness for mandatory variables (Figure 5A). Figure 5B shows a heterogeneous completeness distribution, with many patients between 30% and 60%, primarily corresponding to FREDDEX-imported records that were prefilled according to the information available in BaMaRa and are progressively enriched through routine curation. Approximately one-quarter (456/1649, 27.7%) of patients have fully completed (100%) the mandatory data.

Data quality analysis (Figure 5A) identified inconsistencies in 7% (110/1649) of patients. These mainly reflect logical inconsistencies related to incomplete records rather than incorrect data entry, such as missing dependent variables.

Slightly less than 40% (605/1649, 36.7%) of patients had at least one associated image (Figure 5A). Image completeness varied across modalities (Figure 5C), reaching nearly 50% (767/1649, 465% and .768/1649, 46.6%) for fundus photography versus approximately 30% (455/1649, 27.6% and 451/1649, 27.3%) .for OCT. This difference reflects both routine clinical practice and operational constraints, as OCT and autofluorescence are acquired less systematically and imaging integration into France Cohortes requires additional workflows outside SKEZIA.

### Lessons Learned

#### Operational Bottlenecks and Challenges

##### Institutional and Regulatory Workstreams

**Figure 6.**
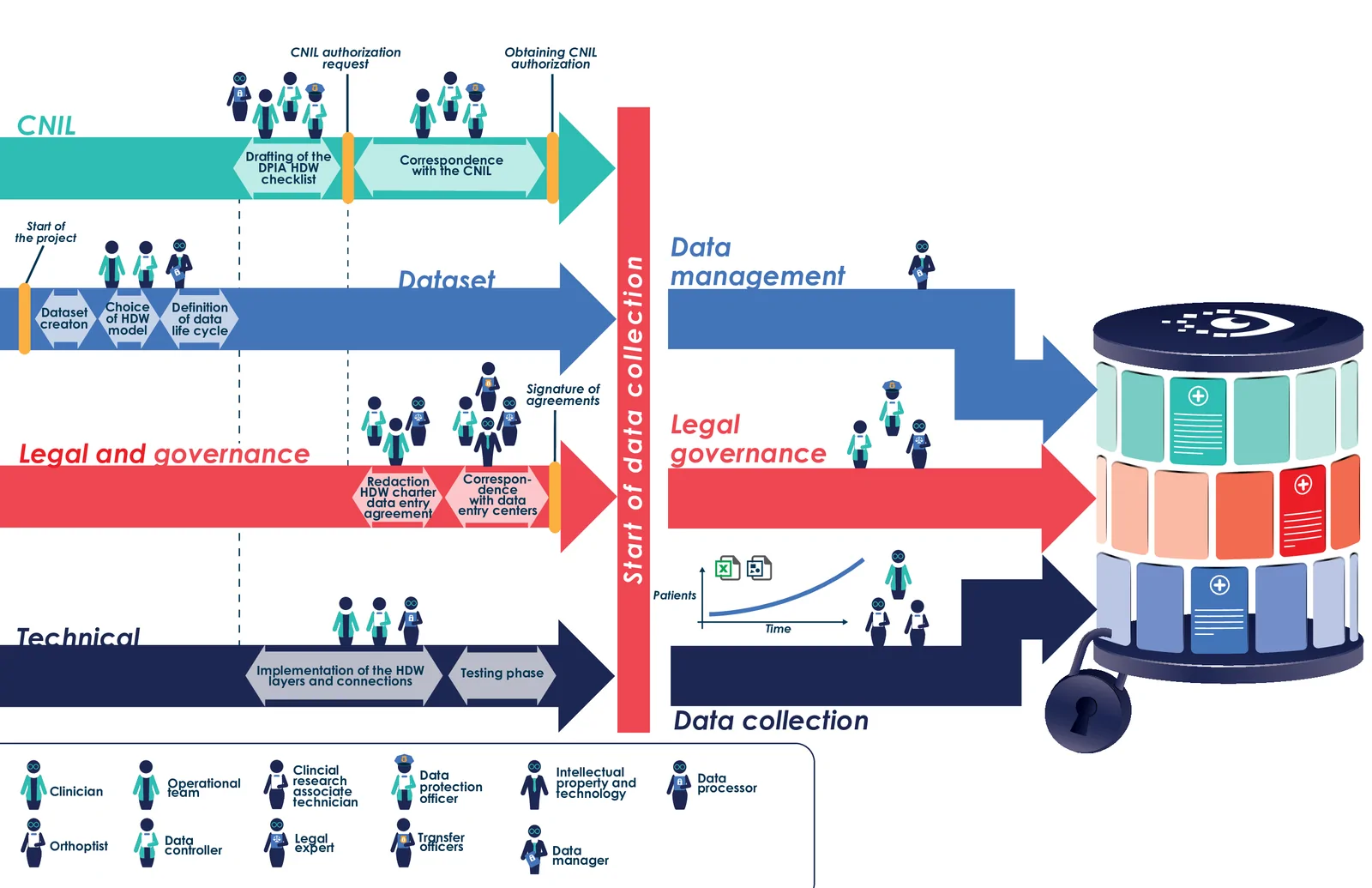
Parallel timelines leading to the creation of a health data warehouse (HDW) through 4 key areas: regulatory (involving the French regulation bodies), data-related (dataset definition), legal (establishment of legal documentation), and technical (implementation of tools for data processing). The key actors are also displayed. CNIL: French data protection authority; DPIA: data protection impact assessment.

Setting up FREDD within the highly demanding CNIL HDW framework introduced significant decision-making latency due to the need to coordinate numerous institutional stakeholders. To mitigate these administrative hurdles, our experience demonstrates that regulatory, legal, and technical workstreams must progress concurrently rather than sequentially to maintain project momentum (Figure 6).

Beyond central governance, preparing the database deployment across multiple hospital networks requires substantial time. In this new AI era, healthcare institutions have become acutely aware of the strategic value of clinical and imaging data for training AI models. Consequently, negotiating data-sharing agreements is no longer an administrative formality; it involves protracted, detailed discussions to protect institutional assets and ensure clear returns on investment.

Crucially, these combined delays create an operational bottleneck where project staff are fully funded but data collection cannot yet begin, risking budgetary exhaustion prior to final deployment. Project planning must therefore build strict financial and temporal flexibility into timelines to absorb the impact of these modern negotiation landscapes.

##### Human Resources, User Engagement, and Tool Flexibility

The implementation of FREDD underscored an acute scarcity of specialized personnel—such as data protection officers, health data lawyers, data engineers, and clinical research technicians—within French public health care institutions, highlighting a systemic need to professionalize these hybrid roles. This resource scarcity directly challenged on-the-ground user engagement.

To prevent the platform from being perceived as an administrative encumbrance by clinical teams, securing long-term medical commitment was essential. We overcame this by implementing role-specific onboarding protocols, providing continuous on-site support, simplifying user interfaces, and demonstrating the direct scientific value of each module to build investigator trust.

Furthermore, data collection tools must be engineered with sufficient flexibility to align with diverse real-world clinical routines, as rigid architectures inevitably cause friction and workflow disruptions. Establishing a continuous feedback loop between participating pilot sites and the central operational team proved vital for iteratively refining the eCRF, the FREDDEX parsing tool, and automated quality control modules.

### Key Success Factors

#### Rapid Early Outcomes and Onboarding Dynamics

Once the regulatory and technical foundations were finalized, platform adoption across the pilot centers was remarkably swift, reflecting strong initial engagement by the clinical teams. This effective uptake is illustrated by a sharp, nonlinear increase in data collection, creating an unprecedented high-density repository for REDs.

Because strict regulatory frameworks and data quality pipelines were fully embedded into the infrastructure during the “Build” phase, ongoing operational efforts can now shift from structural setup to continuous adaptation. Future resources will focus entirely on microadjustments driven by emerging research queries and evolving data regulations, ranging from national compliance updates to broader European alignments.

#### Downstream Research and Public Health Impact

By systematically aggregating data from geographically dispersed expert centers, the FREDD repository is designed for continuous expansion, steadily increasing the statistics essential for RED research. This centralized, harmonized approach has already unlocked initial opportunities for rapid scientific use. The immediate availability of curated data has supported the launch of early-stage initiatives, including preliminary multicenter epidemiological analyses and pilot AI-driven predictive modeling projects, which are currently ongoing.

Ultimately, by bridging this longitudinal clinical depth with a fully compliant regulatory framework, FREDD delivers a uniquely valuable asset for both clinical research and public health impact. It serves as a functional example for how modern HDWs can structurally transform multicenter collaboration, accelerating discoveries in fields where data scarcity remains the primary bottleneck.

A comprehensive synthesis of the key barriers, enabling factors, resource requirements, and actionable recommendations derived from this implementation process is provided in Multimedia Appendix 6.

## Discussion

### Scalability, Imaging Standardization, and AI Readiness

Expanding FREDD from 5 pilot centers to a nationwide infrastructure will introduce organizational and technical challenges. Our implementation experience suggests that clinical data harmonization is unlikely to become the main bottleneck, as the common data model and standardized collection procedures proved transferable across centers despite differing local workflows. Instead, scalability will primarily depend on the storage and processing of ophthalmic imaging, the largest component of the dataset volume. Progressive onboarding should nevertheless allow infrastructure scaling over time.

Beyond structured clinical variables, ophthalmic imaging presents additional challenges for AI applications. Although the current workflow enables secure image integration, image standardization and normalization still require validation through ongoing research projects. Future work will therefore focus on refining these processes while developing scalable, multiexpert annotation frameworks for rare ocular manifestations.

Moreover, as FREDD expands beyond inherited retinal disorders, the data model will require disease-specific extensions. Its modular architecture, however, allows new ophthalmology-specific modules to be integrated while preserving the shared rare disease data core.

### Long-Term Sustainability and Financial Models

Sustaining FREDD beyond its initial funding period presents significant financial and organizational challenges. While maintaining the France Cohortes hosting environment and the SKEZIA platform generates recurring costs, human resources—including project management, data curation, regulatory oversight, quality control, and local data collection—represent the largest long-term expenditure.

As disease-specific HDWs currently receive limited governmental support for routine operations, FREDD is developing a data access and cost-recovery framework aligned with the HDH Starter Kit for HDWs [42]. This framework relies on three principles:

1. Differentiated access models—separating cost-recovery mechanisms for academic and industrial users
2. Public-interest preservation—ensuring that cost-recovery and industrial partnerships do not compromise the infrastructure’s scientific mission
3. Support for participating centers—reinvesting cost-recovery fees into local data collection, storage, and curation activities

As disease-specific HDWs remain relatively new infrastructures, sustainable funding models are still evolving. FREDD’s long-term viability will therefore depend on iterative refinement based on operational experience, user demand, and evolving national and European funding priorities.

### Conclusions and Future Perspectives

The FREDD initiative demonstrates that strict adherence to the CNIL HDW framework from the very outset does not hinder technological innovation. Instead, this early regulatory integration serves as a foundational catalyst for institutional trust—providing the exact governance guarantees that health care networks now demand before releasing their high-value datasets into collaborative research ecosystems.

While establishing this framework required substantial upfront organizational and financial investments over several years, the rapid inclusion of 1649 patients within the first year of active data collection signals that FREDD has successfully moved past its implementation phase.

At this stage, the critical challenge shifts from infrastructure setup to scientific activation. The true value of the warehouse will now depend on the systematic launch of multicenter research projects, the deployment of AI-driven clinical models, and its deep integration into national and European data ecosystems. By strengthening direct interoperability with cross-border infrastructures such as the REDgistry, FREDD serves as a scalable research engine whose long-term impact will transform collaborative research in the field of rare diseases.

## Supplementary material

10.2196/99206 Multimedia Appendix 1 Detailed technical architecture, data security, and storage workflows.

10.2196/99206 Multimedia Appendix 2 Structure of the FREDD (French Rare Eye Disease Database) dataset and distribution of variables by category.

10.2196/99206 Multimedia Appendix 3 Detailed composition of complex categories of FREDD (French Rare Eye Disease Database) dataset.

10.2196/99206 Multimedia Appendix 4 Detailed imaging processing pipeline.

10.2196/99206 Multimedia Appendix 5 Chronological timeline of FREDD (French Rare Eye Disease Database) infrastructure phases, regulatory milestones, and deployment.

10.2196/99206 Multimedia Appendix 6 Synthesis of the FREDD (French Rare Eye Disease Database) implementation framework: barriers, enablers, resources, and recommendations.

10.2196/99206 Checklist 1 i-CHECK-DH checklist.

## Abbreviations

BaMaRa: Base Maladies Rares
BNDMR: Banque Nationale de Données Maladies Rares
CNIL: Commission nationale de l’informatique et des libertés
DICOM: Digital Imaging and Communications in Medicine
DPIA: Data Protection Impact Assessment
eCRF: electronic case report form
EHDS: European Health Data Space
ERN: European Reference Network
FREDD: French Rare Eye Disease Database
FREDDIn: FREDD Insights
FSMR: Filières de santé maladies rares
GDPR: General Data Protection Regulation
HDH: Health Data Hub
HDW: health data warehouse
HGNC: HUGO Gene Nomenclature Committee
HPO: Human Phenotype Ontology
INSERM: Institut national de la santé et de la recherche médicale
IRD: inherited retinal dystrophy
OCT: optical coherence tomography
PNMR: National Plan for Rare Diseases
RED: rare eye disease
RP: retinitis pigmentosa
