# Supplementary material for: Building a National Interoperable Rare Eye Disease Data Warehouse: Methodological Framework and Implementation Report From the French Rare Eye Disease Database (FREDD) Initiative
Source: JMIR Med Inform. 2026 Aug 25;14:e99206. doi: 10.2196/99206 (PMC13507042; doi:10.2196/99206)
Supplement: Supplementary file 1 [file medinform-v14-e99206-s001.docx]

# Detailed technical architecture, data security, and storage workflows

## Data collection

To ensure high-quality, research-ready datasets, FREDD utilizes a custom electronic case report form (e-CRF) built from scratch using SKEZIA. This e-CRF incorporates automated logic checks, mandatory fields, and validation workflows to maintain internal consistency and harmonization across participating centers. Furthermore, SKEZIA exposes an API that enables seamless integration with external systems.

To protect patient privacy, the resulting data stream undergoes early pseudonymization, with identifying and clinical information strictly compartmentalized and encrypted using dedicated keys. The correspondence tables linking patient identities to pseudonyms are stored in a physically and logically isolated environment, independently encrypted from the main clinical dataset.

## Secure data transfer and processing

All clinical, genetic, and imaging datasets are transferred from the collection layer to France Cohortes via FileSender, a secure solution compliant with national regulatory requirements for high-volume data batches. Currently, this transfer process operates on a semi-manual basis due to API development constraints between SKEZIA and FileSender, with progressive automation planned for future phases. To safeguard data integrity in the interim, local centers follow strict standard operating procedures: structured data are packaged into a single CSV file and images into a single ZIP archive to minimize manipulation and facilitate verification. Upon receipt, all packages undergo central data-management review to immediately correct anomalies. Once validated, structured data are ingested into the central repository, while imaging data undergo specific processing pipelines.

## Data storage, access control and secondary use

All data are hosted within secure, isolated virtual environments in France Cohortes, featuring encrypted storage, action traceability, strict access controls, and redundant backup systems. Permissions are regulated by a formalized data access matrix based on the principle of least privilege: participating centers can exclusively access their own data, while the FREDD data manager holds read-and-write rights solely for non-identifiable datasets. Secondary data use requires FREDD governance committee approval; upon validation, researchers are granted access within a dedicated, project-specific secure workspace.
