# Supplementary material for: Building a National Interoperable Rare Eye Disease Data Warehouse: Methodological Framework and Implementation Report From the French Rare Eye Disease Database (FREDD) Initiative
Source: JMIR Med Inform. 2026 Aug 25;14:e99206. doi: 10.2196/99206 (PMC13507042; doi:10.2196/99206)
Supplement: Supplementary file 2 [file medinform-v14-e99206-s002.docx]

| **FREDD dataset category** | **Total variables** | **Mandatory variables** |
| --- | --- | --- |
| Legal | 7 | 2 |
| Administrative | 18 | 9 |
| Status | 4 | 1 |
| Clinical diagnosis^a^ | 61 | 13 |
| Genetic diagnosis^b^ | 196 | 54 |
| Disease history^c^ | 28 | 13 |
| Visual acuity examination | 29 | 19 |
| Visual field examination | 22 | 21 |
| Treatments | 6 | 0 |
| Disability | 7 | 0 |
| Relevant medical history | 31 | 0 |
| Clinical protocols^d^ | 13 | 0 |
| Image deposit | 25 | 21 |

Table S1: Structure of the FREDD dataset and distribution of variables by category.

**a** Clinical diagnosis includes one primary diagnosis and one optional additional diagnosis.
**b** Genetic diagnosis includes up to three genes and up to three variants per gene, recorded for one primary diagnosis and one optional additional diagnosis.
**c** Disease history is collected for one primary diagnosis and one optional additional diagnosis.
**d** Clinical protocol information can be recorded for up to three protocols per patient.
