# Supplementary material for: Building a National Interoperable Rare Eye Disease Data Warehouse: Methodological Framework and Implementation Report From the French Rare Eye Disease Database (FREDD) Initiative
Source: JMIR Med Inform. 2026 Aug 25;14:e99206. doi: 10.2196/99206 (PMC13507042; doi:10.2196/99206)
Supplement: Supplementary file 3 [file medinform-v14-e99206-s003.docx]

| **Category** | **Subcomponent** | **Variables** | **Mandatory variables** |
| --- | --- | --- | --- |
| Clinical diagnosis | Diagnosis-related | 30 | 6 |
| Clinical diagnosis | Other variables | 1 | 1 |
| Genetic diagnosis | Diagnosis-related | 98 | 27 |
| Genetic diagnosis | Gene-related | 3 | 2 |
| Genetic diagnosis | Variant-related | 9 | 2 |
| Genetic diagnosis | Other variables | 8 | 3 |
| Disease history | Diagnosis-related | 13 | 6 |
| Disease history | Other variables | 2 | 1 |
| Clinical protocols | Protocol-related | 4 | 0 |
| Clinical protocols | Other variables | 1 | 0 |

Table S2: Detailed composition of complex categories of FREDD dataset.
