# Supplementary material for: Building a National Interoperable Rare Eye Disease Data Warehouse: Methodological Framework and Implementation Report From the French Rare Eye Disease Database (FREDD) Initiative
Source: JMIR Med Inform. 2026 Aug 25;14:e99206. doi: 10.2196/99206 (PMC13507042; doi:10.2196/99206)
Supplement: Supplementary file 4 [file medinform-v14-e99206-s004.docx]

# Detailed imaging processing pipeline

Collecting these unstructured images across multiple centers poses specific challenges due to heterogeneous acquisition devices, varied file formats (DICOM, proprietary formats, JPEG, PNG), diverse resolutions, and inconsistent metadata schemas. Furthermore, these images contain inherently identifiable features. Because they must preserve fine-grained structural information for AI-based and quantitative analyses, traditional anonymization methods cannot be applied.

To overcome these challenges, initial imaging datasets are processed following a standardized pipeline. This pipeline combines technical standardization, privacy protection, and multi-level quality control through four successive steps:

1. Image harmonization: Images are standardized via resizing and RGB color encoding to facilitate research reuse.
2. Metadata reconstruction and DICOM generation: A new DICOM object is generated from scratch for every single image across all modalities and lateralities, combining the processed image with essential research metadata and the patient pseudonym, while strictly excluding all direct identifiers.
3. Hybrid identification detection and human verification: Dedicated software screens image content to automatically flag and isolate files with embedded text or report headers, which are then manually inspected by authorized operators to guarantee absolute pseudonymization.
