# Supplementary material for: Building a National Interoperable Rare Eye Disease Data Warehouse: Methodological Framework and Implementation Report From the French Rare Eye Disease Database (FREDD) Initiative
Source: JMIR Med Inform. 2026 Aug 25;14:e99206. doi: 10.2196/99206 (PMC13507042; doi:10.2196/99206)
Supplement: Supplementary file 5 [file medinform-v14-e99206-s005.docx]

| **Date** | **Phase / Milestone** | **Description** |
| --- | --- | --- |
| **October 2022** | **Project launch** | Official start of the **RaReTiA** project. |
| **December 2022** | **Regulatory initiative** | Submission of the Data Protection Impact Assessment (DPIA). |
| **April 2024** | **Legal authorization** | Official **CNIL authorization** obtained for FREDD. |
| **September 2024** | **Governance inception** | Establishment of the governance structure and approval of the first version of the **FREDD Charter**. |
| **March 2025** | **Early technical deployment** | FREDD data collection layer achieves full operational capacity and compliance. |
| **March 2025** | **Data collection launch** | Patient recruitment and structured clinical data collection begin within the 5 SENSGENE pilot centers. |
| **June 2025** | **Governance formalization** | Official signing of the HDW Charter. |
| **November 2025** | **Full infrastructure compliance** | Complete France Cohortes infrastructure reaches full compliance with all CNIL technical and organizational requirements. |
| **November 2025** | **Multimodal data integration and data migration** | Launch of imaging data integration into the centrally secured storage environment and migration of all data to the France Cohortes infrastrcuture |

Table S3: Chronological timeline of FREDD infrastructure phases, regulatory milestones, and deployment.
