# Supplementary material for: Building a National Interoperable Rare Eye Disease Data Warehouse: Methodological Framework and Implementation Report From the French Rare Eye Disease Database (FREDD) Initiative
Source: JMIR Med Inform. 2026 Aug 25;14:e99206. doi: 10.2196/99206 (PMC13507042; doi:10.2196/99206)
Supplement: Supplementary file 6 [file medinform-v14-e99206-s006.docx]

| **Framework dimension** | **Key barriers and challenges** | **Enabling factors and successes** | **Resource requirements** | **Practical recommendations for future adopters** |
| --- | --- | --- | --- | --- |
| **Regulatory and legal** | High decision-making latency due to highly demanding CNIL HDW framework; complex, multi-stakeholder institutional coordination. | Early regulatory anticipation and alignment with national strategies; data compliance used as a structural backbone rather than a constraint. | Dedicated health data experts; data protection officers | Parallelize workstreams: run regulatory submissions, legal contracts, and technical setups concurrently rather than sequentially; anticipate long institutional negotiation timelines. |
| **Institutional and governance** | Lengthy negotiations of data collection agreements; hospitals' high awareness of data value (especially for AI), requiring proven return of interest. | Strong initial engagement and shared vision within the SENSGENE network; rapid proof of recruitment viability (1649 patients in Year 1). | Formal governance bodies with clear data reuse access right; structured data-access committees ensuring the active participation of wiling data-providing centers. | Incentivize local centers: Guarantee transparent data governance, active representation, and clear co-authorship/academic credit rules from day one. |
| **Human resources and engagement** | Acute scarcity of specialized profiles in public healthcare; risk of clinical teams perceiving data entry as an administrative burden. | Stepwise, role-specific onboarding protocols; strong trust built through on-site technical and regulatory support. | Dedicated data health experts for HDW supervision; dedicated clinical research technicians; project management personnel. | **Build budget flexibility:** Allocate structural funding to absorb project extensions and prevent staffing gaps: professionalize and support hybrid technical/clinical roles within hospital networks. |
| **Technical and operational** | Rigid architectures mismatching local workflows; high storage/processing demands driven by heavy imaging data volume. | Agile interface design and flexible architecture; automated embedded quality control and logical inconsistency checks. | Scalable cloud hosting infrastructure; iterative, user-driven e-CRF updates. | Establish continuous feedback loops: Maintain close communication between local centers and the core technical team to iteratively refine data-import pipelines.    • Plan for progressive storage scaling. |
| **Data activation and future readiness** | Challenges in image standardization and normalization: high difficulty in generating reproducible, high-quality multi-expert annotations for AI. | Clear data processing and standardization procedures; rapid data readiness for pilot AI models and epidemiological studies. | Multi-expert annotation tools; seamless interoperability tools | **Design for interoperability:** Align data models and cost-recovery frameworks with national and emerging international standards to facilitate inner and cross-border cohort aggregation. |
| International Generalizability | High regulatory and security constraints specific to the French CNIL HDW framework. | Achieving compliance under one of Europe’s strictest regulatory standards validates the core model. | Institutional state funding and specialized human expertise (legal, security, and data handling). | **Delineate transferable components:** Treat specific French tools (like BaMaRa/BNDMR) as local context. However, the core **three-layer data isolation architecture**, the **parallelized workflow structuring** (technical-legal-regulatory), and the **governance committee design** can be directly replicated as a validated, maximum-security blueprint under GDPR, UK, or North American frameworks. |

Table S4: Synthesis of the FREDD implementation framework: barriers, enablers, resources, and recommendations.
