# Supplementary material for: Building a National Interoperable Rare Eye Disease Data Warehouse: Methodological Framework and Implementation Report From the French Rare Eye Disease Database (FREDD) Initiative
Source: JMIR Med Inform. 2026 Aug 25;14:e99206. doi: 10.2196/99206 (PMC13507042; doi:10.2196/99206)
Supplement: Supplementary file 7 [file medinform-v14-e99206-s007.docx]

**iCHECK-DH Checklist – FREDD Implementation Report**

**Manuscript:** Building a national interoperable rare eye disease data warehouse: methodological framework and implementation report from the FREDD initiative
**Reference guideline:** Perrin Franck et al., J Med Internet Res. 2023;25:e46694. PMID: 37163336.

| **Section** | **Item** | **Mandatory** | **Manuscript location** | **Response / checklist entry** |
| --- | --- | --- | --- | --- |
| Title | 1. Title | M | Title | The title identifies the work as an implementation report: “Building a national interoperable rare eye disease data warehouse: methodological framework and implementation report from the FREDD initiative.” |
| Abstract | 2. Abstract | M | Abstract | The abstract is structured as Background, Objectives, Methods, Results, and Conclusions. It reports the implementation setting, five pilot centers, 1649 patients, more than 10,000 ophthalmic images, and the main early operational outcomes. |
| Introduction | 3. Context | M | Introduction – Context; The French rare disease ecosystem; European integration: ERN-EYE and REDgistry | The manuscript describes the French national rare disease context, the SENSGENE network, the national BNDMR/BaMaRa ecosystem, ERN-EYE and REDgistry, the regulatory environment, and the initial implementation context. The implementation is described as a national infrastructure with a phased deployment initially involving five expert centers. |
| Introduction | 4. Problem Statement | M | Introduction – Identified gap | The manuscript explicitly identifies the lack of a dedicated, interoperable research-oriented health data warehouse for rare eye diseases and the resulting fragmentation between routine clinical data collection, national registry data, and research reuse. |
| Introduction | 5. Similar Interventions | M | Introduction – Identified gap; Methods – Aims and objectives / Interoperability | No comparable implementation linking BaMaRa/BNDMR to a dedicated rare eye disease research health data warehouse was identified. FREDD was developed specifically to fill this gap. Its design builds on the existing BaMaRa/BNDMR and SDM-MR ecosystem while adding a dedicated multimodal rare eye disease research infrastructure and interoperability layer. |
| Methods | 6. Aim and Objectives | M | Methods – Aims and objectives | The manuscript defines four implementation objectives: establish a nationally coordinated RED repository; enable multicenter research, cohort building, and longitudinal studies; facilitate interoperability with BNDMR, ERN-EYE, and the EHDS; and support new diagnostic and therapeutic strategies. |
| Methods | 7. Implementation Strategy | M | Methods – Blueprint summary | The implementation strategy is described through the CNIL HDW reference framework. |
| Methods | 8. Technical Design | M | Methods – Technical design; Data; Interoperability; Multimedia Appendix 1 and Multimedia Appendix 4 | The technical design is described in detail, including the three-layer HDW architecture (collection, storage/processing, and research). Detailed technical architecture, data security, and storage workflows are provided in Multimedia Appendix 1; the imaging workflow is detailed in Multimedia Appendix 4. |
| Methods | 9. Target | M | Methods – Target | FREDD is designed for patients affected by rare eye diseases nationwide. The initial implementation uses a controlled phased deployment across five SENSGENE expert centers, initially focusing on inherited retinal dystrophies, particularly retinitis pigmentosa. |
| Methods | 10. Data | M | Methods – Data; Regulatory aspects, governance and phased deployment; Ethics Approval; Data Availability | The manuscript describes the data lifecycle, data minimization, structured and imaging data, pseudonymization and image de-identification, secure deletion of raw source images, data governance, access controls, research reuse procedures, patient information, GDPR/CNIL compliance, hosting within France Cohortes, and restrictions on individual-level data availability. |
| Methods | 11. Interoperability | M | Methods – Interoperability | The manuscript describes technical and semantic interoperability with BNDMR/BaMaRa and European infrastructures. It specifies the use of SDM-MR, Orphanet/ORPHA, HPO, HGNC, HGVS, FREDDEX, and the planned REDDEX bridge toward European data models and infrastructures. |
| Methods | 12. Participating Entities | M | Methods – Participating entities; Funding Statement | FREDD is developed by the SENSGENE rare disease network and operates administratively and legally under the responsibility of INSERM as central data controller. Five pilot expert centers are listed individually, and the RaReTiA/ANR France 2030 funding is described. As FREDD is a health data warehouse, the manuscript focuses on data governance and institutional responsibility; intellectual property relating to downstream research projects is outside the scope of this implementation report. |
| Methods | 13. Budget Planning | M | Methods – Budget planning and sustainability | The manuscript reports that FREDD was launched and developed through the RaReTiA project (ANR-21-PMRB-0009), funded under the PNMR3/France 2030 rare disease database call. The dedicated funding covered the Build phase, initial deployment, and beginning of the Run phase, but is time-limited. Exact budget amounts are not reported in the manuscript due to the early phase of the HDW. |
| Methods | 14. Sustainability | M | Discussion – Long-term sustainability and financial models | The manuscript describes the sustainability challenge after the initial RaReTiA funding period, recurring infrastructure and human-resource costs, and the development of a data-access and cost-recovery framework aligned with the Health Data Hub Starter Kit for HDWs. It specifies differentiated access models, preservation of the public-interest mission, support for participating centers, and the need for iterative refinement of the sustainability model. |
| Implementation Results | 15. Coverage | M | Results – Coverage | The implementation covered five participating SENSGENE expert centers in France. During one year of active data acquisition (March 2025 to March 2026), 1649 patients were included and more than 10,000 ophthalmic images were integrated. The manuscript also reports that the 763 patients with retinitis pigmentosa represented 20.3% of the national BNDMR RP cohort. |
| Implementation Results | 16. Outcomes | M | Results – Outcomes | The manuscript reports early implementation outcomes including data completeness, data inconsistency rates, imaging coverage, and the number of patients and images integrated. Approximately 7% of patients had identified inconsistencies, slightly less than 40% had associated imaging, fundus photography completeness approached 50%, and OCT completeness was approximately 30%. |
| Implementation Results | 17. Lessons Learned | M | Results – Lessons learned; Operational bottlenecks and challenges; Key Success Factors | The manuscript reports lessons concerning regulatory and institutional delays, the need to run regulatory, legal, and technical workstreams concurrently, data-sharing negotiations, financial and temporal flexibility, scarcity of specialized profiles, user engagement, continuous site feedback, tool flexibility, and rapid onboarding once regulatory and technical foundations were established. |
| Implementation Results | 18. Unintended Consequences | NM | Not covered |  |
| Discussion | 19. Conclusion | M | Discussion – Conclusion and future perspectives | The conclusion summarizes the main implementation findings, emphasizing the feasibility of deploying a regulated, multimodal rare eye disease health data warehouse, the importance of early regulatory integration, the transition from infrastructure setup to scientific activation, and future integration with national and European ecosystems. |
| General | 20. General | NM | Ethics Approval; Funding Statement; Conflicts of Interest; Data Availability; Acknowledgements | The manuscript reports CNIL authorization, the regulatory framework, funding through ANR/France 2030, conflicts of interest, data availability restrictions, and acknowledgements. The study does not involve a conventional clinical trial registration. |
